# Feasibility of AmbulanCe-Based Telemedicine (FACT) Study: Safety, Feasibility and Reliability of Third Generation In-Ambulance Telemedicine

**DOI:** 10.1371/journal.pone.0110043

**Published:** 2014-10-24

**Authors:** Laetitia Yperzeele, Robbert-Jan Van Hooff, Ann De Smedt, Alexis Valenzuela Espinoza, Rita Van Dyck, Rohny Van de Casseye, Andre Convents, Ives Hubloue, Door Lauwaert, Jacques De Keyser, Raf Brouns

**Affiliations:** 1 Department of Neurology, Universitair Ziekenhuis Brussel, Brussels, Belgium; 2 Center for Neurosciences (C4N), Vrije Universiteit Brussel (VUB), Brussels, Belgium; 3 Flanders District of Creativity, Leuven, Belgium; 4 Department of Emergency Medicine, Universitair Ziekenhuis Brussel, Brussels, Belgium; 5 Research Group on Emergency and Disaster Medicine (ReGEDiM), Vrije Universiteit Brussel (VUB), Brussels, Belgium; 6 Department of Neurology, University Medical Center Groningen, University of Groningen, Groningen, The Netherlands; The University of Queensland, Australia

## Abstract

**Background:**

Telemedicine is currently mainly applied as an in-hospital service, but this technology also holds potential to improve emergency care in the prehospital arena. We report on the safety, feasibility and reliability of in-ambulance teleconsultation using a telemedicine system of the third generation.

**Methods:**

A routine ambulance was equipped with a system for real-time bidirectional audio-video communication, automated transmission of vital parameters, glycemia and electronic patient identification. All patients ( ≥18 years) transported during emergency missions by a Prehospital Intervention Team of the Universitair Ziekenhuis Brussel were eligible for inclusion. To guarantee mobility and to facilitate 24/7 availability, the teleconsultants used lightweight laptop computers to access a dedicated telemedicine platform, which also provided functionalities for neurological assessment, electronic reporting and prehospital notification of the in-hospital team. Key registrations included any safety issue, mobile connectivity, communication of patient information, audiovisual quality, user-friendliness and accuracy of the prehospital diagnosis.

**Results:**

Prehospital teleconsultation was obtained in 41 out of 43 cases (95.3%). The success rates for communication of blood pressure, heart rate, blood oxygen saturation, glycemia, and electronic patient identification were 78.7%, 84.8%, 80.6%, 64.0%, and 84.2%. A preliminary prehospital diagnosis was formulated in 90.2%, with satisfactory agreement with final in-hospital diagnoses. Communication of a prehospital report to the in-hospital team was successful in 94.7% and prenotification of the in-hospital team via SMS in 90.2%. Failures resulted mainly from limited mobile connectivity and to a lesser extent from software, hardware or human error. The user acceptance was high.

**Conclusions:**

Ambulance-based telemedicine of the third generation is safe, feasible and reliable but further research and development, especially with regard to high speed broadband access, is needed before this approach can be implemented in daily practice.

## Introduction

Telemedicine refers to the use of information and communication technology to provide healthcare services to individuals who are at distance from a specialized healthcare provider [1]. It is currently mainly applied as an in-hospital service that brings medical expertise to underserved geographical areas [2]–[8].

The first generation telemedicine systems were ‘point-to-point’ models over landlines, confining health care providers to fixed workstations within hospitals [9]. The involvement of the World Wide Web (WWW) heralded the second generation of telemedicine, allowing teleconsultations to be conducted from anywhere at any time [9]. Third generation telemedicine (telemedicine 3.0) leverages mobile broadband connectivity to expedite prehospital teleconsultations between patients in moving ambulances and remote healthcare providers. Prehospital transmission of ECG is widely used to reduce the time-to-treatment and to improve outcomes in patients with acute myocardial infarction [10]–[12]. Remote assisted abdominal ultrasound [13] and virtual presence of emergency physicians have been investigated to support paramedics in the field [14], [15]. Prehospital telemedicine for stroke promises to be a feasible [16] and reliable [17] solution to reduce door-to-needle times [18].

## Methods

We report on the Feasibility of AmbulanCe-based Telemedicine (FACT) study, which is part of the Prehospital Stroke Study at the Universitair Ziekenhuis Brussel (PreSSUB) [17], [19]. The aim of this prospective study is to investigate the safety, the technical feasibility and the reliability of in-ambulance telemedicine using a prototype third generation telemedicine system (PreSSUB 3.0). To avoid treatment delay in emergency settings, informed consent was obtained on opt-out basis, followed by written informed consent from patients or legal representatives after the acute phase. All patients gave written informed consent for participation in the study. The individual in this manuscript has given written informed consent (as outlined in PLOS consent form) to publish these case details. The study protocol and the consent process were approved by the ethics committee of the Universitair Ziekenhuis Brussel (B.U.N. 143201317990) on January 8^th^ 2014. The FACT study was registered at clinicaltrials.gov (NCT02119598). As the study duration was shorter than expected due to high enrolment rates, registration of the study was completed only after patient enrolment was terminated. The authors confirm that all ongoing and related trials for this intervention are registered.

### PreSSUB 3.0 system

**Figure 1:**
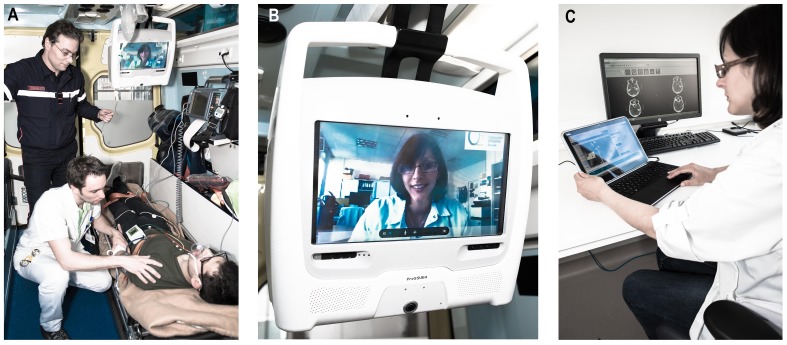
The PreSSUB 3.0 system. The telemedicine device is securely mounted to the ceiling of the ambulance (A) and allows bidirectional audiovisual communication between the patient and the teleconsultant via integration of a microphone, speakers, a screen and a 360° view camera (B). The teleconsultant has mobile access to the telemedicine platform using a lightweight laptop computer with touch screen, integrated microphone, speakers and a webcam (C).

Building on our experience involving in-ambulance telemedicine in healthy volunteers [17], we equipped a routine ambulance used in emergency interventions with a system for real-time bidirectional audio-video communication, allowing virtual face-to-face interaction between patients and teleconsultants (Figure 1). Other functionalities include the automated transmission of vital parameters (heart rate, blood oxygen saturation, systolic and diastolic blood pressure), glycemia, electronic patient identification (eID), electronic reporting and prehospital notification of the in-hospital team by short message service (SMS).

The architecture of the PreSSUB 3.0 device in the ambulance consists of a laptop computer (Dell Latitude XT3, Dell, Round Rock, Texas, USA) operating on 64-bit Windows 7 (Microsoft Corp., Redmond, USA) with touch screen and integrated microphone and loudspeaker, connected to a 360° view Internet protocol camera (Mobotix Q24, Mobotix AG, Winnweiler, Germany) and a 4G router (RUT550 LTE, Teltonika, Vilnius, Lithuania). The camera offers remote digital pan, tilt and zoom (8X) functionalities, as well as user-defined preset commands. Real-time audiovisual and vital data are transmitted over a mobile broadband connection (3G/4G, Belgacom) through a transmission unit in the ambulance and a roof antenna. The video stream is integrated into a telemedicine platform (WiPaM, IXSyS, Hasselt, Belgium) that also hosts the proprietary software for Unassisted TeleStroke Scale (UTSS) assessment. This scale was developed in Dutch, French and English and validated as a rapid tool for assessment of stroke severity through telemedicine, without the assistance of a third party at the patients' bedside [17], [19].

Image size and compression quality of the video image were kept constant during the study at 16 images per second in a standard VGA format of 640×480 pixels. Data privacy was secured by password-protected login, role-based access control, hypertext transfer protocol secure encryption, and transfer through a virtual private network. Wireless point-of-care devices for measurement of blood pressure and glycemia (Clever Chek TD3250-C, RDSM, Hasselt, Belgium), blood oxygen saturation (CMS 60 Pulse Oximeter, Contec TM, Qinhuangdao, China) and an eID card reader, were incorporated in the system. Prehospital notification was initiated by the teleconsultant by sending an automated SMS message from the telemedicine platform to the cell phones of in-hospital caregivers, informing them that an electronic report of the prehospital consultation was available on a designated, password protected website.

To guarantee mobility and to facilitate 24/7 availability, the teleconsultants used lightweight laptop computers (Dell XPS, Dell, Round Rock, Texas, USA) with touch screen, integrated microphones, loudspeakers and webcams and a 64-bit Windows 8 or Windows 8.1 operating system (Microsoft Corp., Redmond, USA). To access ambulance data and to control the camera in real-time they used secured WiFi networks or a 4G modem (Huawei E398, Huawei Technologies Co, Shenzhen, China) and a standard WWW browser (Opera 19.0, Opera Software ASA, Oslo, Norway). To obtain bi-directional audiocommunication, an Internet phone service was used (Skype TM, version 6/14/59.104, Skype/Microsoft Corp. Redmond, USA).

### Study participants

All patients ( ≥18 years) transported during emergency missions by a Prehospital Intervention Team (PIT) of the Universitair Ziekenhuis Brussel from February 13th to February 28th 2014 were eligible for inclusion in the study. The PIT consists of two Emergency Medical Technicians (EMT) and one Certified Emergency Nurse (CEN) or Critical Care Registered Nurse (CCRN). Secondary patient transports or inter-hospital transfers were not included. Patients were investigated by one of three teleconsultants (LY, RJVH and RB) who had at least five years of clinical experience in neurological emergencies. The study did not interfere with PIT dispatches, which occurred according to local guidelines, nor with the standing orders. Activation of the device was easily done by the nurse in the ambulance via one-button activation. All CEN and CCRN nurses (n = 16) followed a three-year general training program, a one-year training program in emergency care and additional field experience training. Prior to the start of the FACT study, they received training on the use of the PreSSUB 3.0 system.

A control population was included, consisting of all patients transported by the PIT of the UZ Brussel during emergency missions between January 1st and January 31th 2014.

### Data collection and analysis

The bandwidth of the mobile connection and the total amount of data transfer were measured during the teleconsultations using an open-source bandwidth monitor (BitMeter OS v0.7.5, Copyright 2011 Rob Dawson) and screen recordings of all teleconsultations were obtained (BB Flashback 4.1, Blueberry software Ltd., Birmingham, United Kingdom). Any issue with hardware, software, connectivity or safety was recorded.

Data on patient characteristics (demographics, prehospital and in-hospital diagnosis) and prehospital time intervals were retrieved from the reports of the teleconsultants, PIT reports and the medical hospital records. For the control populations, data on patient characteristics (demographics, prehospital Glasgow Coma Scale and prehospital diagnosis) and intervention characteristics (interventions outside office hours, PIT intervention time) were retrieved from the PIT reports and the medical hospital records.

The quality of the audio-video connection and the user-friendliness of the system was rated by the teleconsultants and the nurses using Likert-scales [20]. Telephone-based or real-life debriefings were conducted immediately after the teleconsultation when necessary and all nurses were interviewed in person after the study to obtain detailed feedback.

Statistical analysis was performed using SPSS statistics version 22.0 (SPSS, Chicago, IL, USA). Technical data (bandwidth and data transfer) and patient demographics (age) were not normally distributed and are therefore presented as medians (interquartile ranges, IQR). Other data are presented as success rates (number of successful registrations divided by the number of attempts ×100). The agreement between the prehospital telediagnosis and the final in-hospital diagnosis was evaluated using the proportion of overall agreement and κ statistics.

## Results

### Patient population

**Figure 2:**
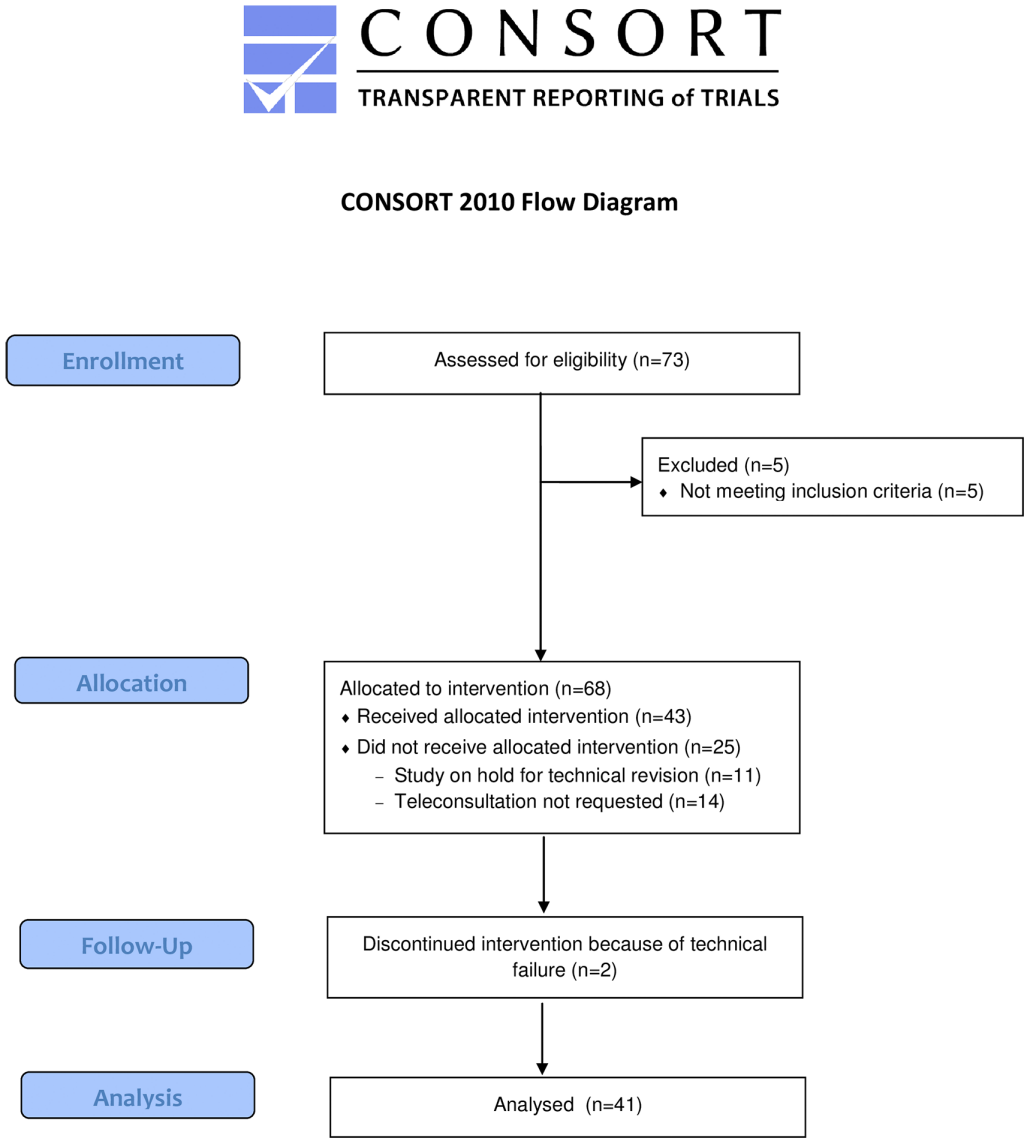
Flow diagram of the FACT study.

During the study, the PIT of the Universitair Ziekenhuis Brussel transported 73 patients of which 68 patients met the inclusion criteria. Forty-three attempts were made to perform a prehospital teleconsultation. In 11 cases no attempt was made, as the telemedicine system was being revised for technical reasons and in 14 cases, teleconsultation was not requested by the PIT. This occurred mainly because of patient characteristics (aggression, psychiatric disease, labor) (Figure 2).

Prehospital teleconsultation was obtained in 41 out of 43 cases (success rate, 95.3%). Failures resulted from technical problems; in one case the device was not fully functional because it initiated in safe mode after forced shut down during the previous session. In another case, the failure resulted from a power issue as the battery of the system had not been recharged. Bidirectional audio-video communication was established in 39 cases. In 2 cases, only unidirectional video communication from the ambulance to the teleconsultant was obtained because of low bandwidth. A video fragment of a prehospital teleconsultation is available as supplemental material (Video S1).

### Patient data and medical data

The patients' median age was 57.0 years (IQR, 38.5–80.5). Twenty males (48.8%) and 21 females (51.2%) participated. Teleconsultation was performed in the language preferred by the patient, which was French in 33 cases (80.5%) and Dutch in 8 cases (19.5%). No significant differences in patient demographics were found between the study group and the control group (Table 1). None of the eligible patients refused participation.

**Table 1:** Patient and intervention characteristics.*

	FACT	Control group	*P* value
	*(n = 41)*	*(n = 134)*	
**Patient characteristics**			
Male gender^§^	20 (48.8%)	57 (42.5%)	*0.299*
Age (years) ^#^	57.0 (38.5–80.5)	65.0 (37.8–78.0)	*0.905*
Prehospital Glasgow Coma Scale score^#^	15 (15–15)	15 (14–15)	*0.514*
Prehospital diagnosis PIT^§^			
Cardiac arrest	0 (0.0%)	1 (0.7%)	*1.000*
Serious trauma	4 (9.8%)	6 (4.5%)	*0.247*
Respiratory distress	5 (12.2%)	11 (8.2%)	*0.535*
Acute coronary syndrome	0 (0.0%)	4 (3.0%)	*0.574*
Stroke	3 (7.3%)	9 (6.7%)	*1.000*
Intoxication	3 (7.3%)	10 (7.5%)	*1.000*
Other	26 (63.4%)	93 (69.4%)	*0.566*
**Intervention characteristics**			
Outside office hours^§^	28 (68.3%)	71 (53.0%)	*0.059*
PIT intervention time (minutes) ^#^	36 (29–51)	36 (28–47)	*0.625*

* Data given as number (percentage) or as median (interquartile range). ^§^Fisher's exact test. ^#^Mann-Whitney U test.

Abbreviations: FACT, Feasibility of AmbulanCe-based Telemedicine; PIT, Prehospital

Intervention Team.

Patient identification was attempted if the patient's eID card was available (n = 19). In 3 cases, the eID data was not transmitted by the system (success rate, 84.2%). Registration of the blood pressure and the heart rate via the PreSSUB system was performed during 33 sessions. Successful registration of the systolic and diastolic blood pressure was obtained in 78.7%. Registration of the heart rate was successful in 84.8%. All failures resulted from data transmission issues between the device and the telemedicine platform. Measurement of blood oxygen saturation was attempted in 31 cases, with successful registration in 80.6%. One failure resulted from battery failure of the device, all other failures resulted from data transmission issues. In 8 cases, a non-PreSSUB device (Lifepak 15, Physio-control, Redmond, USA) was used for registration of blood pressure, heart rate and blood oxygen saturation. In 2 patients, blood oxygen saturation could not be measured due to agitation. Glycemia was successfully registered in 64.0% (n = 25). One device error occurred, other failures resulted from data transmission issues. In 6 cases a non-PreSSUB device (Accu-Chek Performa, Roche, Basel, Switzerland) was used for registration of glycemia. Data obtained with non-PreSSUB devices were transmitted verbally to the teleconsultant when possible.

Blood pressure abnormalities were noted in 77.5% of available registrations and included systolic hypertension (SBP ≥140 mmHg) in 58.8%, diastolic hypertension (DBP ≥90 mmHg) in 45.4% and diastolic hypotension (DBP ≤60 mmHg) in 12.1% [21], [22]. Six patients presented with a hypertensive urgency (SBP >180 or DBP >120) [22]. Abnormalities in heart rate occurred in 37.1%, with tachycardia (HR >100 bpm) in 25.7% and bradycardia (HR <60 bpm) in 11.4% [23]. Three patients presented with a relevant cardiac arrhythmia (7.3%), involving atrial fibrillation with ventricular response >150 bpm in 2 patients and a cardiac arrest requiring cardiopulmonary resuscitation in one patient. Only in the latter case assistance of an emergency physician was called on-site. Hypoxemia (spO2 <95%) was present in 20.7% and 3 patients presented with severe hypoxemia (spO2 <90%). Dysglycemia was present in 16.7%, all involving hyperglycemia (glycemia >200 mg/dl).

In patients with an emergency call for suspicion of an acute neurological disease, the UTSS [17], [19] was used to identify stroke and to evaluate the stroke severity.

In 7 patients (17.1%), a neurological condition was suspected based on information from the emergency medical services (EMS) dispatch, with suspicion of epileptic seizure, acute stroke and coma in respectively 3, 2 and 2 patients. In 6 out of the 7 cases, an attempt was made to perform an UTSS assessment. In 2 patients (33.3%), the assessment was completed before arrival at the hospital. Only partial evaluation was possible in 4 cases (66.6%), due to technical issues related to connectivity in one case and due to patient characteristics related to the disease (vomiting, coma, aphasia) in 3 cases.

A preliminary prehospital diagnosis, based on the results of the clinical examination and the vital parameters, was formulated by the teleconsultants in 37 cases (90.2%) (Table 2). Failure to obtain a prehospital diagnosis was the result of connectivity issues with permanent or temporary disconnection during the teleconsultation in 4 cases.

**Table 2:** Detailed overview of the prehospital telemedicine diagnoses and the final in-hospital diagnose.

	Prehospital telemedicine diagnosis	Final in-hospital diagnosis
**Neurological disease**	8 (19.5%)	8 (19.5%)
Stroke	5 (12.2%)	3 (7.3%)
Other	3 (7.3%)	5 (12.2%)
**Non-neurological disease**	29	33 (80.5%)
Trauma	10 (24.4%)	10 (24.4%)
Respiratory disease	8 (19.5%)	9 (22.0%)
Gastro-intestinal disease	3 (7.3%)	4 (9.8%)
Intoxication	3 (7.3%)	3 (7.3%)
Acute pain	2 (4.9%)	3 (7.3%)
Labor	2 (4.9%)	1 (2.4%)
Dysglycemia	1 (2.4%)	1 (2.4%)
Vascular disease	0 (0.0%)	1 (2.4%)
Other	0 (0.0%)	1 (2.4%)
**Unknown**	4 (9.8%)	0 (0.0%)

There was a high degree of agreement between the prehospital telediagnosis and the final inhospital diagnosis. The proportion of overall agreement and the κ statistic for distinguishing neurological disease from non-neurological disease were 0.98 and 0.92, respectively (*P*<0.001). All patients with in-hospital diagnosis of stroke were correctly identified prehospitally (proportion of overall agreement, 0.93; κ statistic, 0.54; *P*<0.001). One out of 43 patients in the FACT study suffered from acute ischemic stroke and was eligible from treatment with intravenous recombinant tissue plasminogen activator (IV r-tPA). This patient was correctly identified as a candidate for thrombolytic treatment during the prehospital teleconsultation.

Communication of the prehospital report to the in-hospital team was attempted in 38 cases. The reports contained the patient's identity, vital parameters, the results of the UTSS when relevant and additional patient information and observations obtained by the teleconsultant. Two failures occurred due to software or human error (success rate, 94.7%). The in-hospital team was notified of the patient's arrival and the availability of a written report via SMS in 37 cases (90.2%). All SMS messages arrived within 60 seconds after activation from the telemedicine platform by the teleconsultant. No SMS was sent in 4 cases because no relevant information had been obtained during the teleconsultation (n = 3) or due to human error (n = 1).

### Intervention characteristics

The median PIT intervention time (defined as the time between PIT departure and arrival of the patient in the hospital) was 36 minutes (IQR, 29–51 minutes) in the study group. The median duration of the teleconsultation was 10 minutes (IQR, 7–13 minutes). No significant differences in intervention characteristics were found between the study group and the control group (Table 1).

### Technical data

#### Mobility and connectivity of teleconsultants

Twenty-eight teleconsultations (68.3%) were conducted outside office hours (between 6 p.m. and 8 a.m). Twenty-six teleconsultation sessions (63.4%) were performed by the teleconsultants while being outside of the hospital. In 25 cases, the teleconsultant was at home (61.0%) and in 1 case (2.4%) at another location. Internet connection was obtained by the teleconsultant through WiFi in 33 sessions (80.5%) and through 4G in 8 sessions (19.5%). No signal losses occurred during teleconsultations using 4G internet access, but this system was only used outside office hours.

#### Bandwidth

**Figure 3:**
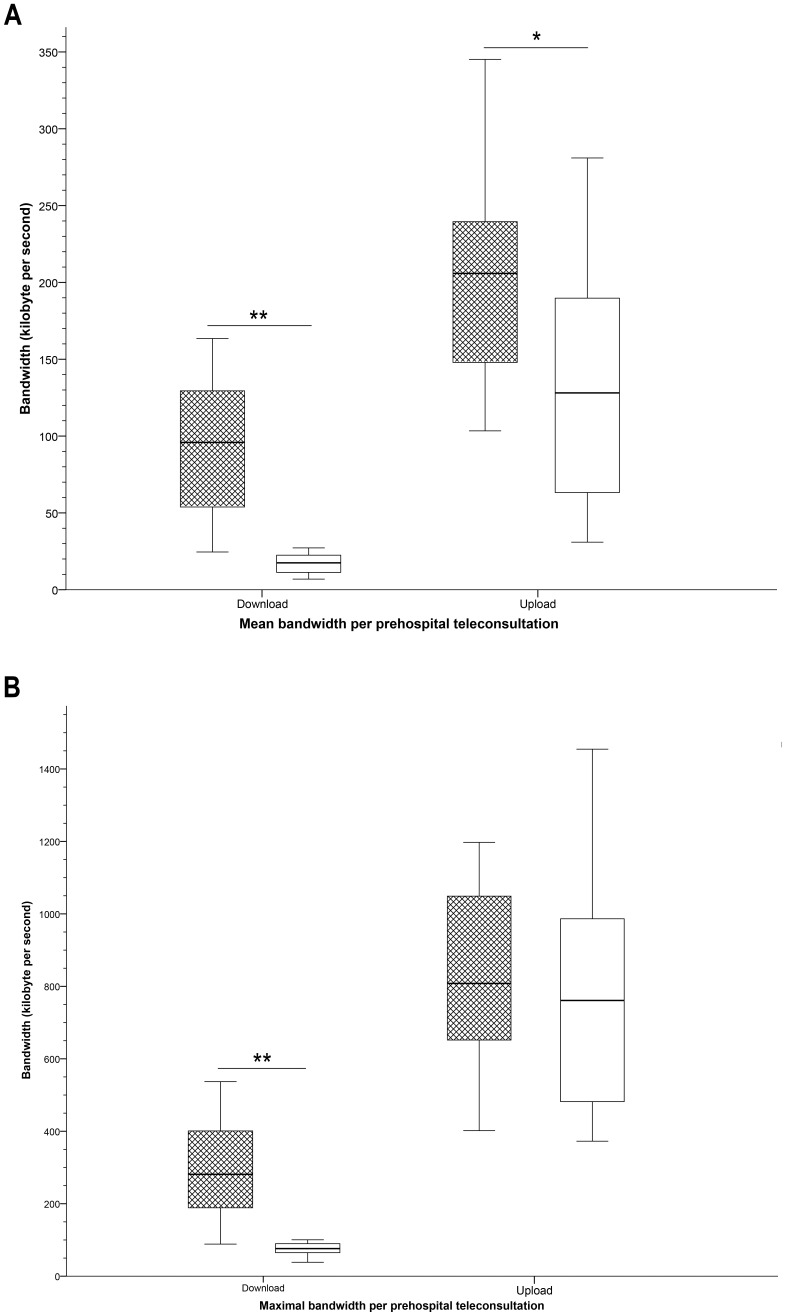
Box-and whisker plots demonstrating bandwidth per prehospital teleconsultation. Box-and whisker plots demonstrating mean (**A**) and maximal (**B**) bandwidth per prehospital teleconsultation for download (from the ambulance to the teleconsultant) and for upload (from the teleconsultant to the ambulance). Hatched boxes represent teleconsultations outside of office hours; white boxes teleconsultations during office hours. Significant differences are indicated with * (*P*<0.05) or with ** (*P*<0.001).

Mean and maximal upload speeds (from the teleconsultant to the ambulance) and download speeds (from the ambulance to the teleconsultant) were registered per teleconsultation, as were the data transfers to and from the ambulance were registered per teleconsultation (Table 3). These results are according to expectations as the file transfer load from the ambulance is much larger than the file transfer load towards the ambulance. The bandwidth varied in a diurnal pattern (Figure 3). Compared to office hours, the mean and maximal download speed was significantly higher outside of office hours (*P*<0.001 for both). The mean upload speed was significantly lower during office hours compared to outside office hours (*P* = 0.02).

**Table 3:** Bandwidth and data transfer during prehospital teleconsultation*.

	Upload	Download
	(from the teleconsultant to the ambulance)	(from the ambulance to the teleconsultant)
**Mean speed** (kB/s)	163 (139–221)	54 (19–113)
**Maximal speed** (kB/s)	812 (641–1060)	167 (84–377)
**Total data transfer** (MB)	102 (74–180)	35 (13–57)

* Data given as median (interquartile range).

Abbreviations: kB/s: kilobytes per second; MB: megabyte.

#### Signal loss

**Figure 4:**
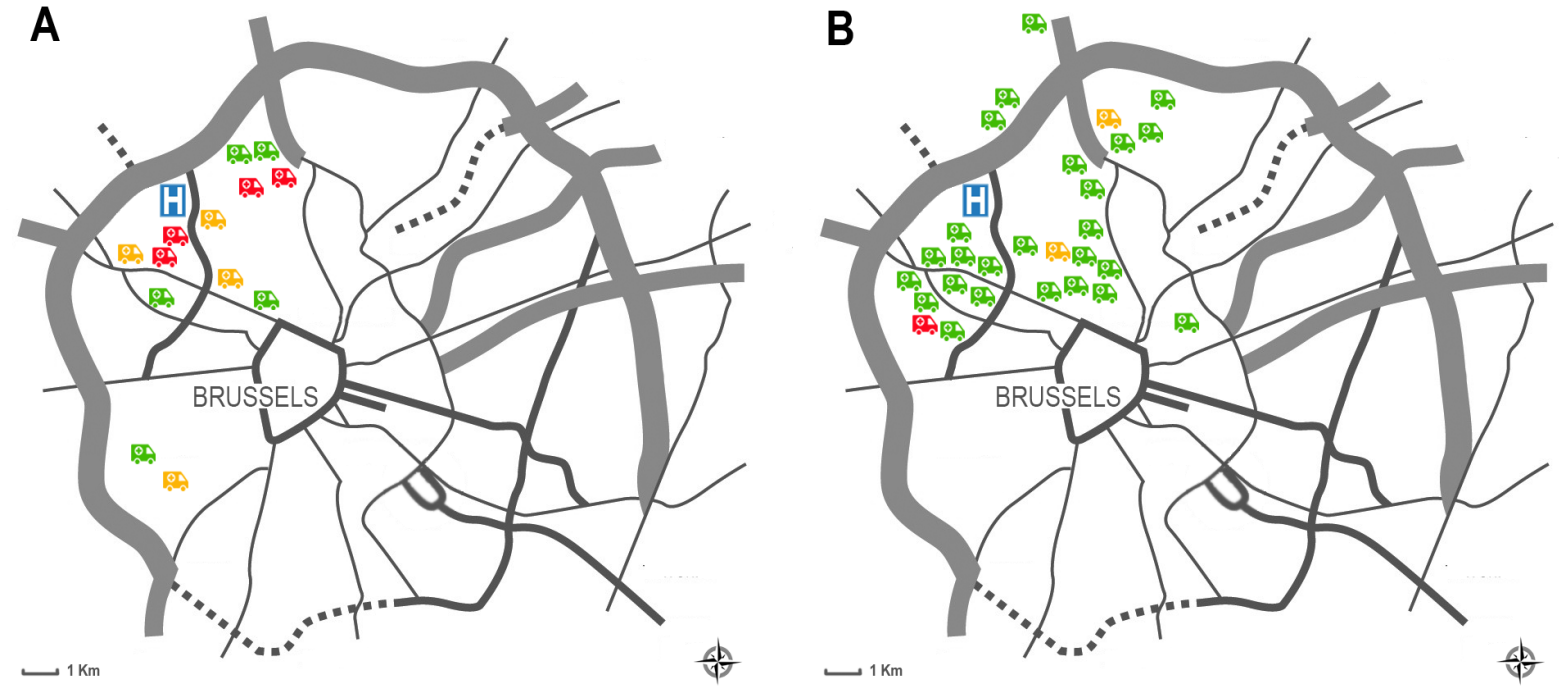
Map of Brussels indicating connectivity during prehospital telemedicine consultations. Map of Brussels indicating the location of the Universitair Ziekenhuis Brussel (H) and the patient locations according to connectivity during prehospital telemedicine consultations (no signal loss: green ambulance; transient signal loss: yellow ambulance; permanent signal loss: red ambulance) during office hours (**A**) and outside office hours (**B**).

Thirty teleconsultations were performed without any signal loss (success rate, 73.2%). Transient signal loss occurred during 6 teleconsultation sessions (14.6%), of which 2 were performed outside office hours and 4 during office hours (Figure 4). The time before the connection was re-established varied from 38 seconds to 5 minutes and 47 seconds. Permanent signal losses occurred in 5 teleconsultations (12.2%), all of which were performed during office hours. Mean up- and download speeds were significantly lower in teleconsultations with signal loss compared to those without signal loss (*P* = 0.021 and *P*<0.001, respectively).

### Safety and acceptability

No adverse events or safety issues occurred during the study. Patient acceptability of ambulance based telemedicine consultation was reflected by the fact that none of the patients refused teleconsultation. Using Likert scale assessments (File S1), the video quality was rated as ‘Good’ (median score  = 4; IQR, 4–4) by the teleconsultants and the audio quality was rated as ‘Speech easily understandable, little noise or distortion’ (median score  = 4; IQR, 3–4). The overall user-friendliness of the teleconsultation system as rated by the teleconsultant was ‘Good’ (median score  = 4; IQR, 4–4). The teleconsultants rated the quality of the report as ‘Good’ (median score  = 4; IQR 4–4). In 84.2% of cases the teleconsultants judged that the consultation provided added value to routine prehospital practice.

The video quality was rated as ‘Good’ (median score  = 4; IQR, 3–4) by the PIT and the audio quality was rated as ‘Good’ (median score  = 4; IQR, 4–5). The overall user-friendliness of the teleconsultation system as rated by the PIT was ‘Good’ (median score  = 4; IQR, 3.5–4). The PIT nurses rated the registration and transmission of vital parameters as 'Good' (median score  = 4; IQR, 2.5–5).

## Discussion

This study is the first to evaluate the safety, the technical feasibility and the clinical reliability of third generation prehospital telemedicine during primary emergency transport by a PIT. No safety issues occurred and clinically useful information was obtained in over 90% of in-ambulance teleconsultations, despite connectivity issues. Most problems were caused by unstable bandwidth of the 3G/4G mobile network. This technical issue has previously been described [15], [16], [24], [25] and several solutions are being explored. The TeleBAT project [24], [26] relied on mobile technology with limited bandwidth and unstable connectivity, which completely precluded real-time conferencing. Liman *et al.* [25] deemed 3G connectivity to be unacceptable for clinical use because adequate assessment was possible in only 40% of the scenarios. In the Aachen project [16], [27], broadband communication via parallelized data channels was used as an attempt to remediate unstable connectivity and dropouts. However, technical instability of video transmission during transport remained a problem. Improvement was obtained in their follow-up study using 3^rd^ generation mobile networks and roof antennas [15]. In a previous pilot study with healthy volunteers, 4G technology showed promising results [17], but prioritized access to high speed broadband networks for medical services is currently not available and competition with other users therefore remains an issue.

As expected in a densely populated urban area, bandwidth varied in a diurnal and a geographic pattern with the highest level of connectivity outside office hours, relating to weaker competition with other users for high speed broadband access. Also, more requests for prehospital telemedicine were registered in this time period, which could partially be explained by the reduced accessibility to general practitioners in this time frame. This finding accentuates the advantages of telemedicine 3.0, allowing teleconsultations to be performed outside office hours without confining the teleconsultants to a teleconference room inside the hospital. Mobility of teleconsultants strengthens the scalability and the implementation of prehospital telemedicine and is an important leverage in terms of user-friendliness, 24/7 availability of highly qualified caregivers and possibly also from an economic viewpoint.

The accuracy of prehospital telediagnosis was high, both for distinguishing neurological vs. non-neurological conditions and for identifying possible stroke patients. Moreover, the only candidate for IV r-tPA was correctly identified through prehospital teleconsultation.

Furthermore, the general acceptance of the system was high, both by teleconsultants and the PIT nurses. This is reflected by the high rates of the system activation (75.4%) by the nurses and the Likert-scale based assessment for user-friendliness. Prehospital teleconsultation did not cause delay of the PIT intervention.

This study's main limitations include the small sample size, the short study duration and the observational design. Although the FACT study reports on the largest patient cohort evaluated by prehospital telemedicine so far, the risk of selection bias should be acknowledged. Furthermore, this is the first study evaluating patients outside office hours and comparing them to matched controls. The observational design of this feasibility study did not allow interventions by the teleconsultants and thus precludes evaluation of possible effects of prehospital telemedicine on the quality of medical care and the outcome. However, the safety, feasibility, and the number of abnormal vital parameters registered during teleconsultation, further substantiate the potential of this approach to improve patient care. Finally, prehospital teleconsultation was not formally evaluated from the patients' perspective, but the fact that none of the patients refused participation is encouraging.

## Conclusions

Prehospital telemedicine of the third generation is a safe and technically feasible solution for real-time bidirectional audio-video communication between patients in a moving ambulance and remote healthcare providers. Our results are promising, but further research and development is warranted before this technique can be implemented in daily practice. Technical issues, especially with regard to high speed broadband access remain to be resolved. Prioritized connection for medical services seems an achievable solution in short-term.

## Supporting Information

Video S1 **Video fragment of a prehospital teleconsultation.** The video shows a screen capture from a teleconsultants' laptop computer illustrating a sample of a prehospital teleconsultation, displaying real-time bidirectional audiovisual interaction between the patient and the Prehospital Intervention Team nurse in the ambulance and the remote teleconsultant. The left hand side of the telemedicine platform shows the video stream from the 360° view Internet protocol camera with zooming capability. The right hand side of the screen features the proprietary software for assessment of the Unassisted TeleStroke Scale (in French, as this was the patient's preferred language). The lower part displays the information transferred from the patient's electronic identity card (i.e. name (anonymized), age and gender) and key vital parameters (systolic and diastolic blood pressure, heart rate and blood oxygen saturation). The conversations between the patient, the nurse and the teleconsultant are conducted in French and Dutch. Subtitles are provided at the bottom of the screen. Text in italics refers to voice coming from the ambulance (black for the patient; blue for the nurse). Regular white text indicates voice from the teleconsultant.
(MP4)

File S1 **Likert scales.**
(DOCX)
