# Supplementary material for: Feasibility of AmbulanCe-Based Telemedicine (FACT) Study: Safety, Feasibility and Reliability of Third Generation In-Ambulance Telemedicine
Source: PLoS One. 2014 Oct 24;9(10):e110043. doi: 10.1371/journal.pone.0110043 (PMC4208882; doi:10.1371/journal.pone.0110043)
Supplement: File S1 — Likert scales. (DOCX) [file pone.0110043.s002.docx]

**File S1: Likert scales**

*Likert scale for evaluation of delivered audio quality (DAQ), scored both by teleconsultants and nurses.*

| **DAQ** | **Delivered Audio Quality** |
| --- | --- |
| **5** | Perfect. No distortion or noise discernible. |
| **4** | Speech easily understandable. Little noise or distortion. |
| **3** | Speech understable with slight effort. Requires occasional repetition due to noise or distortion. |
| **2** | Speech understable with considerable effort. Requires frequent repetition due to noise or distortion. |
| **1** | Unusable. Speech present but not understable. |

*Likert scale for evaluation of video quality in absolute category ratings (ACR), scored both by teleconsultants and nurses.*

| **ACR** | **Video quality** |
| --- | --- |
| **5** | Very good |
| **4** | Good |
| **3** | Barely acceptable |
| **2** | Poor |
| **1** | Very poor |

*Likert scale for evaluation of overall teleconsultation quality in absolute category ratings (ACR), scored both by teleconsultants and nurses.*

| **ACR** | **Overall quality of the teleconsultation** |
| --- | --- |
| **5** | Very good |
| **4** | Good |
| **3** | Barely acceptable |
| **2** | Poor |
| **1** | Very poor |

*Likert scale for evaluation of the quality of the transmitted report in absolute category ratings (ACR), scored by the teleconsultants.*

| **ACR** | **Quality of the patient report** |
| --- | --- |
| **5** | Very good |
| **4** | Good |
| **3** | Barely acceptable |
| **2** | Poor |
| **1** | Very poor |

*Likert scale for evaluation of the quality of the transmission of vitals in absolute category ratings (ACR), scored by the nurses.*

| **ACR** | **Transmission of vitals** |
| --- | --- |
| **5** | Very good |
| **4** | Good |
| **3** | Barely acceptable |
| **2** | Poor |
| **1** | Very poor |
